# Adipose-derived stromal cells in regulation of hematopoiesis

**DOI:** 10.1186/s11658-020-00209-w

**Published:** 2020-03-05

**Authors:** Jing Zhang, Yunsheng Liu, Wen Yin, Xingbin Hu

**Affiliations:** 1 Department of Transfusion Medicine, Xijing Hospital, Xi’an, 710032 China; 2 Department of Rocket Force Medicine, Third Military Medical University, Chongqing, 400038 China

**Keywords:** Mesenchymal stromal cells, Hematopoiesis, Adipose-derived stromal cells, Therapy, Stem cells

## Abstract

Over the past decade, mesenchymal stromal cells (MSCs) found in the bone marrow microenvironment have been considered to be important candidates in cellular therapy. However, the application of MSCs in clinical settings is limited by the difficulty and low efficiency associated with the separation of MSCs from the bone marrow. Therefore, distinct sources of MSCs have been extensively explored. Adipose-derived stromal cells (ASCs), a cell line similar to MSCs, have been identified as a promising source. ASCs have become increasingly popular in many fields, as they can be conveniently extracted from fat tissue. This review focuses on the properties of ASCs in hematopoietic regulation and the underlying mechanisms, as well as the current applications and future perspectives in ASC-based therapy.

## Background

Hematopoiesis occurs mainly in the bone marrow (BM) of adult mice and humans [1], which makes the microenvironment indispensable for the maintenance of hematopoietic stem cells (HSCs) [2, 3]. Mesenchymal stromal cells (MSCs) have been identified as essential components of the HSC niche. MSCs are able to produce cytokines such as stem cell factor (SCF), macrophage colony-stimulating factor (M-CSF), stromal cell-derived factor 1 (SDF-1), and angiopoietin 1; and adhesion molecules and extracellular matrix (ECM) proteins such as vascular cell adhesion molecule 1 (VCAM-1), fibronectin, and various selectins, which thus provide support in the self-renewal, differentiation, and homing of HSCs [4, 5]. In addition, MSCs can differentiate into adipocytes, osteoblasts, and chondrocytes to meet the needs of tissue damage repair [6]. Although the mechanisms are not fully understood, MSC-based therapy has been applied in clinical trials, achieving curative outcomes in certain disorders [7]. However, with a relatively lower amount of MSCs being obtained from the BM, MSCs in other tissues or organs are being urgently explored.

### General properties of adipose-derived stromal cells

The isolation procedure and cytological characterization of adult human adipocyte precursors from adipose tissue were studied in the early 1970s [8]. These cells were extracted from diverse anatomical sites, such as superficial abdominal areas, the upper arm, and inguinal and trochanteric areas [9, 10]. Several similar names were given to cells isolated from adipose tissue in different studies, such as adipose-derived adult stem cells, adipose stromal cells, adipose mesenchymal stem cells, preadipocytes, or processed lipoaspirate cells. In order to eliminate this discrepancy, the International Fat Applied Technology Society arrived at a consensus that any plastic-adherent, stable-doubling, and multipotent population of cells derived from lipoaspirate can be referred to as adipose-derived stromal cells (ASCs) [11]. The current procedure to isolate ASCs via liposuction surgery is well controlled and minimally invasive. The number of ASCs found in adipose tissue is notably higher than that of MSCs in bone marrow (BM-MSCs) at the same tissue volume [12]. Studies have revealed that ASCs were more easily obtainable than MSCs derived from the BM.

Zuk and colleagues first identified the multilineage differentiation character of ASCs in 2001 [13]. ASCs were characterized as one kind of adult stem cells, owing to their pluripotent but restricted differentiation ability. In general, culture expanded ASCs are positive for markers such as CD13, CD29, CD44, CD90 and CD105, while they lack hematopoiesis-related markers such as CD14, CD19, CD45, CD106 and HLA-DR [14, 15]. In addition, ASCs have the potential to secrete cytokines and chemokines, such as SCF, granulocyte colony-stimulating factor (G-CSF), interleukin 6 (IL-6), and tumor necrosis factor alpha (TNF-α). ASCs can give rise to multilineage descendants, including adipocytes, osteoblasts, and chondrocytes [16, 17]. Moreover, ASCs secrete adipose-specific proteins, such as leptin and adipsin [18, 19], which are not shared with BM-MSCs (Table 1).

**Table 1:** Comparison between ASCs and BM-MSCs

		ASCs	BM-MSCs	Reference
Source		Adipose tissue	Bone marrow	[20]
Surface markers	CD13 CD14 CD19 CD29 CD44 CD45 CD49d CD90 CD105 CD106 CD166 HLA-DR	+ - - + + - + + + - + -	+ - - + + - - + + + + -	[12, 14, 15, 21, 22]
Cytokines, chemokines, and specific proteins	SCF G-CSF GM-CSF M-CSF SDF-1 IL-1α IL-6 IL-8 IL-12 TNF-α Leptin Adipsin	+ + + + + - + + - + + +	+ + + + + + + + + + - -	[18, 19, 23]
Differentiation	Adipogenesis Chondrogenesis Osteogenesis Myogenesis Angiopoiesis Neurogenesis	+ + + + + +	+ + + + + +	[16, 17, 20]
Cell yield		0.5 × 10^4^ - 2 × 10^5^ cells per gram	1 × 10^3^ cells per mL	[12, 15]
Clinical trials		185	217	
Approved products		Alofisel	Prochymal, Stempeusel, TEMCELL, Hearticellgram-AMI	[24, 25]

The number of registered clinical trials is obtained from the website of the U.S. National Library of Medicine under the terms “adipose-derived stromal/stem cells” and “bone marrow mesenchymal stromal/stem cells” respectively (until September 2019)

### Hematopoiesis-regulating properties of ASCs

The hypothesis that ASCs regulate hematopoiesis like BM-MSCs stems from the fact that they are components of the niche. Several research groups have proved this hypothesis by conducting co-culture assays of ASCs and HSCs in vitro. Nakao et al. proposed that mouse ASCs can improve the expansion and proliferation of CD34^+^ peripheral blood hematopoietic stem/progenitor cells (HSCs/HPCs) as a feeder layer [26]. In the ASC co-culture system, a higher population count of CD34^+^ cells was observed, and the total number of colonies was significantly increased. Nishiwaki et al. drew a similar conclusion using human ASCs from healthy volunteers after a series of ex vivo experiments [27]. In vitro co-culture assays showed that ASCs not only promoted the frequency of CD34^+^ cells, but also yielded more hematopoietic progenitors compared to BM-MSCs. Andreeva et al. also reported that human ASCs supported the expansion of primitive hematopoietic precursors with the CD34^+^CD133^−^ phenotype from the umbilical cord blood [28]. These results suggest that ASCs can act as a feeder layer in the co-culture system and promote CD34^+^ HSC/HPC expansion ex vivo. Besides the supportive capacity of ASCs in the maintenance and proliferation of HSCs, other studies have demonstrated that ASCs help HSCs to differentiate. It was reported that ASCs showed biases in promoting differentiation of HSCs into myeloid and lymphoid lineages, while erythroid progenitors did not change [23]. ASCs particularly helped HSCs to expand myeloid and lymphoid progenitor numbers in vitro, especially granulocytes and their progenitors. However, Zhu et al. suggested that ASCs inhibited the proliferation of erythroleukemia K562 cells in vitro [29]. It has also been proved that ASCs can promote development of megakaryocytes and platelets [30]. The recent studies have not drawn a consistent conclusion on hematopoietic regulation biases of ASCs.

In order to further explore how ASCs support hematopoiesis in vivo, BM transplantations were performed in multiple studies. Nakao and colleagues showed that intra-bone marrow transplantation of ASCs through injection into the tibias facilitated engraftment efficiency and increased homing of donor HSCs [26]. ASCs attracted significantly more Lineage^−^Sca-1^+^c-kit^+^ cells to the BM in comparison with BM-MSCs. This supportive effect in hematopoietic recovery was a result of the dose-dependent effect of ASCs [31], but the effective ASC:HSC ratio differed among several research groups [31, 32]. ASCs were also able to help hematopoietic reconstitution when they were directly transplanted into the BM cavity of wild type and NOD/SCID fatally irradiated mice [26, 27]. Serial transplantations were performed to further elucidate the supportive engraftment effect of ASCs. The results revealed that intravenous co-infusion of ASCs and HSCs improved engraftments in secondary and tertiary transplantation, which indicated that ASCs regulated not only short-term repopulating progenitors but also long-term HSCs [31]. Besides the supportive effect in BM transplantation, the regulatory capacity of ASCs in the differentiation of HSCs was also explored. Lee and colleagues showed that intraperitoneal co-injection with ASCs into NOD/SCID mice promoted the growth of acute lymphoblastic leukemia cells in vivo [33]. Zhang et al. demonstrated that ASCs have the potential to ameliorate platelet recovery in irradiated mice [34]. ASC administration protected BM cells from apoptosis and particularly promoted the frequency of CD41^+^ megakaryocytes within 21 days after irradiation. Thus, these studies indicate that ASCs can support the expansion and specific differentiation of HSCs in vivo. However, the regulatory effects of ASCs on the downstream progenitors of HSCs, including common lymphoid progenitors, common myeloid progenitors, granulocyte-macrophage progenitors, and erythro-myeloid progenitors, should be explored in future studies. Overall, these results provided evidence that ASCs could facilitate hematopoietic reconstitution in vivo, which was consistent with the regulatory effects of ASCs on HSCs in vitro.

### Mechanisms of ASC-mediated regulation in hematopoiesis

**Fig. 1:**
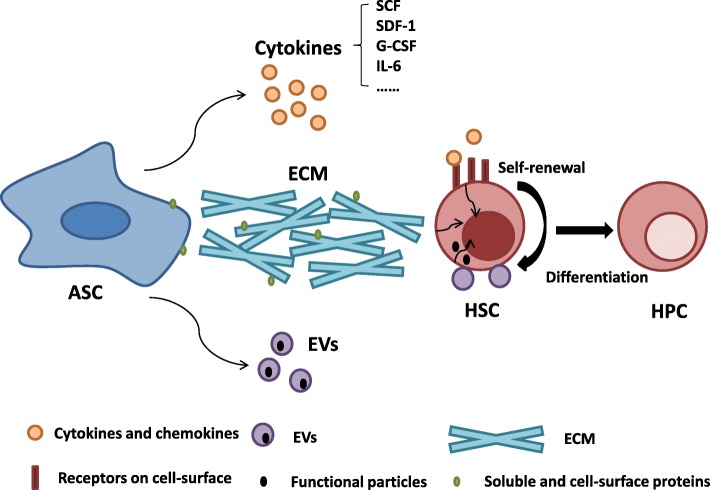
Mechanisms of ASC-mediated regulation in hematopoiesis. The secretion of a variety of cytokines and chemokines is believed to be the main mechanism by which ASCs regulate hematopoiesis. The molecules secreted by ASCs (e.g. SCF, SDF-1, etc.) can bind to the receptors on HSCs. The secretion of EVs is another possible mechanism. The signaling molecules inside the lipid bilayer contribute to intercellular communication. ECM mediates cell adhesion and signal transduction. The soluble and cell-surface proteins expressed by ASCs are thought to be involved in ECM remodeling. Several signal pathways can be activated when HSCs are exposed to ASCs, by which ASCs regulate self-renewal, proliferation, and differentiation of HSCs

The inherent mechanisms by which ASCs regulate hematopoiesis attract much attention in order to better understand their properties related to hematopoietic regulation (Fig. 1). It has been widely accepted that ASCs produce a variety of cytokines with different functions that contribute to the quiescence and bias differentiation of HSCs. Retention factors such as SCF and SDF1 helped to maintain self-renewability and proliferation of HSCs, whereas growth factors such as M-CSF, G-CSF, GM-CSF and IL-6 helped progenitors to differentiate into functional blood cells [35]. SDF1, which is highly expressed by ASCs, could bind to the primary physiological receptor CXCR4 on hematopoietic progenitors. The SDF1-CXCR4 axis played an essential role in maintaining quiescence of HSCs [36]. Similarly, other cytokines could communicate with HSCs or more mature progenitors by binding to their specific receptors. Taken together, ASCs exhibited functional properties in hematopoietic regulation by paracrine action.

In addition, mechanisms and signals other than cytokines are also thought to regulate HSCs. The secretion of extracellular vesicles (EVs) is a possible mechanism for ASC-mediated regulation in hematopoiesis. EVs are functional particles with RNAs, lipids, and proteins inside them, wrapped by a lipid bilayer. These vesicle-associated RNAs are thought to be signaling molecules contributing to intercellular communication [37]. Although there is no report on the function of ASC-derived EVs in hematopoiesis, EVs secreted by MSCs have shown therapeutic effectiveness, including treatment for graft-versus-host disease (GVHD), acute kidney injury, and myocardial ischemia [38, 39]. It has also been proposed by several groups that a type of small, non-coding micro-RNA (miRNA) is involved in the post-transcriptional regulation for maintenance and differentiation of stem cells [40, 41]. ASCs could function as a feeder layer in an ex vivo culture with CD34^+^ cells, and thus promote proliferation of HSCs via overexpression of miR-33 and miR-145 and impairment of p53 function [42, 43]. These miRNAs may have an influence on cell cycle, apoptosis, and senescence of HSCs.

ECM was reported to function as a structural scaffold for cell-to-cell communication between HSCs and the microenvironment [44]. ECM mediated cell adhesion and signal transduction of the surrounding cells to provide functional and biochemical support. TGF-β secreted by ASCs was involved in ECM remodeling and collagen deposition, while CD44 expressed on ASCs affected ECM reorganization [45]. It was noted that several signaling pathways (including Notch-Jagged/Delta, MAPK, Wnt, and Jak-Stat) were activated when HSCs were exposed to MSCs [46]. For example, expression of both the Notch ligands on MSCs and the receptors on HSCs increased in the co-culture system. The activation of Notch signals inhibited the differentiation of HSCs. Wnt signals were involved in Notch activation, which made them vital in self-renewal and proliferation of HSCs as well [47, 48]. These signaling pathways on ASCs should be explored further to better understand hematopoiesis.

### Application of ASCs and future perspectives

ASCs are considered to be useful for therapies in diverse diseases. Due to the paracrine function, multilineage differentiation, and immunological benefits, along with advantages of easy extraction and abundance, ASCs have become increasingly popular in cellular therapy. There are 185 clinical trials registered in the U.S. National Library of Medicine thus far (September 2019), which have been conducted in the treatment of cardiovascular diseases, neurological disorders, skeletal and muscle damage, etc. Among the laboratory studies and clinical trials, the most promising utilization of ASCs was in wound healing and tissue repair [49]. It was reported that direct injection of ASCs into the tissue was effective to cure patients with acute myocardial infarction [50], perianal fistula [51], and chronic injury [52], and combining them with bio-materials showed favorable prognosis for bone defects [53, 54]. A new ASC medicine to treat perianal fistulas in patients with Crohn’s disease has been approved by the European Medicines Agency, indicating that ASC products have moved one step forward in clinical application [55]. Moreover, ASC-based therapy has been shown to be effective in the treatment of GVHD [56] and knee osteoarthritis [57] when administered intravenously. The mechanisms underlying these therapeutic effects include the capability of ASCs to differentiate into mesodermal lineages and secrete soluble factors (TGF-β, VEGF, bFGF), which could enhance tissue regeneration or down-regulate the inflammatory response [58, 59].

MSCs can be obtained mainly from adult tissues including BM, adipose tissue, dental pulp, peripheral blood, muscle and skin; and neonatal sources such as umbilical cord blood (UCB), Wharton’s jelly and placenta [20]. Among the various sources, UCB-MSCs and BM-MSCs are more applied in BM transplantation due to their comparative capacity to regulate hematopoiesis. UCB-MSCs are of higher stemness than other types of adult stem cells with a low immune response. The intravenous administration of UCB-MSCs has been used to treat GVHD in clinical settings [60]. It has been reported that UCB-MSCs helped HSC expansion ex vivo and enhanced the engraftment of HSCs as effectively as BM-MSCs in a murine model [61]. These studies indicated that UCB-MSCs can be used as an alternative source from birth-derived tissues in BM transplantation. As reported previously, ASCs share many features with BM-MSCs, but differ in immunophenotype, differentiation potential, transcriptome, proteome, and immunomodulatory activity [62], which may lead to heterogeneity in functions. Accordingly, ASCs promoted engraftment of HSCs more rapidly than BM-MSCs in murine models [26, 27]. Moreover, among MSCs from all other alternative sources, ASCs exhibited stronger immunosuppressive capabilities than BM-MSCs [63–65]. Research from our and other laboratories has shown that MSCs could facilitate engraftment of HSCs and treat steroid-resistant acute GVHD as well [66, 67]. Both BM-MSCs and ASCs have proved to be effective for the prevention and treatment of GVHD, according to the preliminary phase I/II clinical studies [68–70]. These advantages mentioned above make ASCs a promising candidate to promote hematopoietic reconstitution. The BM contains less than 1 × 10^3^ stromal cells per mL, while there are 0.5 × 10^4^–2 × 10^5^ stromal cells per gram of adipose tissue, which indicates an MSC yield of at least 50-fold more cells from fat tissue than from the BM [12]. These readily accessible and abundant cells perfectly meet the requirement for clinical applications in terms of manipulation.

Meanwhile, scientists have explored new frontiers to extend our knowledge on ASC application. A Japanese group applied a specific culture of ASCs with endogenous TPO to obtain platelets [30], which might be a new technology to address platelet shortage if well controlled and fully developed. ASCs have been used in organ reproduction (e.g. cardiac valves, blood vessels, and bones) in combination with 3D printing. This technology has been successful after transplantation in mammals [71–74], which made this extremely promising to repair damaged organs. ASCs are expected to contribute immensely to cell engineering in future and become one of the main sources of MSCs for clinical application in coming decades.

Although the accumulated studies suggest a potential future for the clinical application of ASCs, several questions need to be answered, with regard to donor and tissue selection, as well as the manner of isolation, expansion, preservation, and infusion of ASCs. It is important to determine whether the biological properties of ASCs are dependent on the donor’s age, gender, body mass index (BMI) and health status. A negative correlation was found between BMI of donor and the yield of ASCs per gram [75]. The harvesting site is also believed to affect properties and yield of cells (Table 2). ASCs from the subcutaneous and omental adipose tissue depot showed differences in cell number and proliferation, but the in vitro differentiation capacity was not different [80, 81]. Other studies provided opposite evidence that cells from subcutaneous depots differentiate faster into adipocytes and osteoblasts than those from visceral depots [78, 79]. Studies have demonstrated that ASCs extracted from superficial abdominal areas were advantageous in senescence over the cells from the upper arm and inguinal and trochanteric areas [9, 10]. It has also been reported that ASC yields are much higher from abdominal subcutaneous tissue than hips and thighs [83]. The above observations indicated that ASCs from different sources might have diverse features, and abdomen could be a preferable tissue for harvesting ASCs in clinical trials.

**Table 2:** Comparison between ASCs from subcutaneous and visceral adipose tissues

Harvesting sites		Subcutaneous depots	Visceral depots	Reference
Source		Abdomen, hips, thighs, knees	Intestines, omentum, perirenal region	[76, 77]
Homogeneity		High	Low	[78]
Surface markers	CD13 CD44 CD90	+ + +	+ + +	[78]
Differentiation	Adipogenesis Chondrogenesis Osteogenesis	+ + +	+ + +	[78, 79]
Proliferation		+	+	[80, 81]
Cell yield		High	Low	[81, 82]

High homogeneity indicates that ASCs isolated from subcutaneous depots have spindle-like morphology and uphold their homogeneity in future passages. Both ASCs from subcutaneous and visceral depots can proliferate and give rise to adipocytes, chondrocytes and osteoblasts, but the capacity of proliferation and differentiation might be different. High cell yield indicates that more ASCs can be obtained from subcutaneous adipose tissues, compared to visceral adipose tissues

Protocols for isolation procedures and methods varied across institutions and laboratories. It has been reported that separation methods (mechanical or enzymatic) played an important role in cell number counts, heterogeneity, and differentiation capacity of ASCs. Higher numbers of ASCs can be obtained via a combination of mechanical and enzymatic procedures [84, 85]. There is a lack of standardized protocols currently; hence, ASCs isolated by various research groups may have different characteristics. More evidence should be provided on the evaluation of cell yield, quality, characteristics, and functions of ASCs from different liposuction studies. Meanwhile, it is important to develop methods to improve the quality and quantity of ASCs.

An ex vivo culture is usually needed for most clinical trials and fundamental studies with ASCs. However, the lack of standardized culture conditions may affect the regeneration, proliferation, and differentiation abilities of ASCs. The freshly isolated and expanded ASCs displayed several distinctions in biomarkers, gene expression, and biological properties (e.g. secretion of cytokines) [86]. Previous studies have shown that the expression of cell markers (e.g. CD105) increased during long-term culture, while others (e.g. CD34) decreased [11]. The genetic integrity was preserved with minimal alteration up to five passages [87]. It is vital to obtain primary cells or cultured cells with fewer passages owing to the dynamic phenotype of ASCs in vitro. Although the selection of optimal passages has also been studied [87, 88], the researchers did not report a consistent conclusion. Future studies need to investigate how to isolate, purify, and characterize ASCs in vitro. Standard protocols, dosage, and manner of administration must be established to apply ASC-based therapies to clinical trials.

## Conclusions

ASCs exhibit properties similar to those of BM-MSCs, including secretion of cytokines, multi-differentiation, and immunological benefits, which confer great potential in therapy. Thus, an in-depth investigation of heterogenicity of MSCs revealed that ASCs can be considered as a competitive candidate. Many previous studies have provided evidence on properties of ASCs in hematopoietic regulation; however, there are unresolved issues that need to be addressed before they can be widely utilized in clinical applications.

## Data Availability

Not applicable.

## Abbreviations

ASCs: adipose-derived stromal cells
bFGF: basic fibroblast growth factor
BM: bone marrow
BMI: body mass index
CXCR4: C-X-C chemokine receptor type 4
ECM: extracellular matrix
EVs: extracellular vesicles
G-CSF: granulocyte colony-stimulating factor
GM-CSF: granulocyte-macrophage colony-stimulating factor
GVHD: graft-versus-host disease
HLA-DR: human leukocyte antigen-antigen D related
HSCs: hematopoietic stem cells
HSCs/HPCs: hematopoietic stem/progenitor cells
IL-6: interleukin 6
M-CSF: macrophage colony-stimulating factor
miRNAs: micro-RNAs
MSCs: mesenchymal stromal cells
SCF: stem cell factor
SDF-1: stromal cell-derived factor 1
TGF-β: transforming growth factor beta
TNF-α: tumor necrosis factor alpha
TPO: thrombopoietin
UCB: umbilical cord blood
VCAM-1: vascular cell adhesion molecule 1
VEGF: vascular endothelial growth factor

## References

[1] Höfer T, Busch K, Klapproth K, Rodewald HR (2016). Fate mapping and quantitation of hematopoiesis in vivo. Annu Rev Immunol.

[2] Kfoury Y, Scadden DT (2015). Mesenchymal cell contributions to the stem cell niche. Cell Stem Cell.

[3] Crane GM, Jeffery E, Morrison SJ (2017). Adult haematopoietic stem cell niches. Nat Rev Immunol.

[4] Singer NG, Caplan AI (2011). Mesenchymal stem cells: mechanisms of inflammation. Annu Rev Pathol.

[5] Schepers K, Campbell TB, Passegué E (2015). Normal and leukemic stem cell niches: insights and therapeutic opportunities. Cell Stem Cell.

[6] Dominici M, Le Blanc K, Mueller I, Slaper-Cortenbach I, Marini F, Krause D (2006). Minimal criteria for defining multipotent mesenchymal stromal cells. The international society for cellular therapy position statement. Cytotherapy..

[7] Fajardo-Orduña GR, Mayani H, Montesinos JJ (2015). Hematopoietic support capacity of mesenchymal stem cells: biology and clinical potential. Arch Med Res.

[8] Van RL, Bayliss CE, Roncari DA (1976). Cytological and enzymological characterization of adult human adipocyte precursors in culture. J Clin Invest.

[9] Brayfield CA, Marra KG, Rubin JP (2010). Adipose tissue regeneration. Curr Stem Cell Res Ther.

[10] Schipper BM, Marra KG, Zhang W, Donnenberg AD, Rubin JP (2008). Regional anatomic and age effects on cell function of human adipose-derived stem cells. Ann Plast Surg.

[11] Baer PC (2014). Adipose-derived mesenchymal stromal/stem cells: an update on their phenotype in vivo and in vitro. World J Stem Cells.

[12] Baer PC, Geiger H (2012). Adipose-derived mesenchymal stromal/stem cells: tissue localization, characterization, and heterogeneity. Stem Cells Int.

[13] Zuk PA, Zhu M, Mizuno H, Huang J, Futrell JW, Katz AJ (2001). Multilineage cells from human adipose tissue: implications for cell-based therapies. Tissue Eng.

[14] Yang Y, Chen X, Li F, Chen YX, Gu LQ, Zhu JK (2015). In vitro induction of human adipose-derived stem cells into lymphatic endothelial-like cells. Cell Reprogram.

[15] Yang D, Wang ZQ, Deng JQ, Liao JY, Wang X, Xie J (2015). Adipose-derived stem cells: a candidate for liver regeneration. J Dig Dis.

[16] Girão-Silva T, Bassaneze V, Campos LC, Barauna VG, Dallan LA, Krieger JE (2014). Short-term mechanical stretch fails to differentiate human adipose-derived stem cells into cardiovascular cell phenotypes. Biomed Eng Online.

[17] Witkowska-Zimny M, Walenko K (2011). Stem cells from adipose tissue. Cell Mol Biol Lett.

[18] Zimmerlin L, Park TS, Zambidis ET, Donnenberg VS, Donnenberg AD (2013). Mesenchymal stem cell secretome and regenerative therapy after cancer. Biochimie..

[19] Zimmerlin L, Donnenberg VS, Pfeifer ME, Meyer EM, Péault B, Rubin JP (2010). Stromal vascular progenitors in adult human adipose tissue. Cytometry A.

[20] Berebichez-Fridman R, Montero-Olvera PR (2018). Sources and clinical applications of mesenchymal stem cells: state-of-the-art review. Sultan Qaboos Univ Med J.

[21] Strem BM, Hicok KC, Zhu M, Wulur I, Alfonso Z, Schreiber RE (2005). Multipotential differentiation of adipose tissue-derived stem cells. Keio J Med.

[22] Yañez R, Lamana ML, García-Castro J, Colmenero I, Ramírez M, Bueren JA (2006). Adipose tissue-derived mesenchymal stem cells have in vivo immunosuppressive properties applicable for the control of the graft-versus-host disease. Stem Cells.

[23] Kilroy GE, Foster SJ, Wu X, Ruiz J, Sherwood S, Heifetz A (2007). Cytokine profile of human adipose-derived stem cells: expression of angiogenic, hematopoietic, and pro-inflammatory factors. J Cell Physiol.

[24] Nitkin CR, Bonfield TL (2017). Concise review: mesenchymal stem cell therapy for pediatric disease: perspectives on success and potential improvements. Stem Cells Transl Med.

[25] Patrikoski M, Mannerström B, Miettinen S (2019). Perspectives for clinical translation of adipose stromal/stem cells. Stem Cells Int.

[26] Nakao N, Nakayama T, Yahata T, Muguruma Y, Saito S, Miyata Y (2010). Adipose tissue-derived mesenchymal stem cells facilitate hematopoiesis in vitro and in vivo: advantages over bone marrow-derived mesenchymal stem cells. Am J Pathol.

[27] Nishiwaki S, Nakayama T, Saito S, Mizuno H, Ozaki T, Takahashi Y (2012). Efficacy and safety of human adipose tissue-derived mesenchymal stem cells for supporting hematopoiesis. Int J Hematol.

[28] Andreeva ER, Andrianova IV, Gornostaeva AN, Gogiya BS, Buravkova LB (2018). Evaluation of committed and primitive cord blood progenitors after expansion on adipose stromal cells. Cell Tissue Res.

[29] Zhu Y, Sun Z, Han Q, Liao L, Wang J, Bian C (2009). Human mesenchymal stem cells inhibit cancer cell proliferation by secreting DKK-1. Leukemia..

[30] Ono-Uruga Y, Tozawa K, Horiuchi T, Murata M, Okamoto S, Ikeda Y (2016). Human adipose tissue-derived stromal cells can differentiate into megakaryocytes and platelets by secreting endogenous thrombopoietin. J Thromb Haemost.

[31] Fernández-García M, Yañez RM, Sánchez-Domínguez R, Hernando-Rodriguez M, Peces-Barba M, Herrera G (2015). Mesenchymal stromal cells enhance the engraftment of hematopoietic stem cells in an autologous mouse transplantation model. Stem Cell Res Ther.

[32] Kilinc MO, Santidrian A, Minev I, Toth R, Draganov D, Nguyen D (2018). The ratio of ADSCs to HSC-progenitors in adipose tissue derived SVF may provide the key to predict the outcome of stem-cell therapy. Clin Transl Med.

[33] Lee MW, Park YJ, Kim DS, Park HJ, Jung HL, Lee JW (2018). Human adipose tissue stem cells promote the growth of acute lymphoblastic leukemia cells in NOD/SCID mice. Stem Cell Rev Rep.

[34] Zhang J, Zhou S, Zhou Y, Feng F, Wang Q, Zhu X (2016). Adipose-derived mesenchymal stem cells (ADSCs) with the potential to ameliorate platelet recovery, enhance megakaryopoiesis, and inhibit apoptosis of bone marrow cells in a mouse model of radiation-induced thrombocytopenia. Cell Transplant.

[35] Kapur SK, Katz AJ (2013). Review of the adipose derived stem cell secretome. Biochimie..

[36] Sugiyama T, Kohara H, Noda M, Nagasawa T (2006). Maintenance of the hematopoietic stem cell pool by CXCL12-CXCR4 chemokine signaling in bone marrow stromal cell niches. Immunity..

[37] Hayashi T, Hoffman MP (2017). Exosomal microRNA communication between tissues during organogenesis. RNA Biol.

[38] Rani S, Ritter T (2016). The exosome - a naturally secreted nanoparticle and its application to wound healing. Adv Mater.

[39] Capomaccio S, Cappelli K, Bazzucchi C, Coletti M, Gialletti R, Moriconi F (2019). Equine adipose-derived mesenchymal stromal cells release extracellular vesicles enclosing different subsets of small RNAs. Stem Cells Int.

[40] Stadler B, Ruohola-Baker H (2008). Small RNAs: keeping stem cells in line. Cell..

[41] Stefani G, Slack F (2008). Small non-coding RNAs in animal development. Nat Rev Mol Cell Biol.

[42] Foroutan T, Farhadi A, Abroun S, Mohammad SB (2018). Adipose derived stem cells affect miR-145 and p53 expressions of co-cultured hematopoietic stem cells. Cell J.

[43] Foroutan T (2017). Increased c-myc and miR-33 expression in expanded hematopoietic stem cells cultured on adipose stem cells feeder layer. Int J Organ Transplant Med.

[44] Valtieri M, Sorrentino A (2008). The mesenchymal stromal cell contribution to homeostasis. J Cell Physiol.

[45] Scioli MG, Storti G, D'Amico F, Gentile P, Kim BS, Cervelli V (2019). Adipose-derived stem cells in cancer progression: new perspectives and opportunities. Int J Mol Sci.

[46] Saleh M, Shamsasanjan K, Movassaghpourakbari A, Akbarzadehlaleh P, Molaeipour Z (2015). The impact of mesenchymal stem cells on differentiation of hematopoietic stem cells. Adv Pharm Bull.

[47] Reya T, Duncan AW, Ailles L, Domen J, Scherer DC, Willert K (2003). A role for Wnt signalling in self-renewal of haematopoietic stem cells. Nature..

[48] Campbell C, Risueno R, Salati S, Guezguez B, Bhatia M (2008). Signal control of hematopoietic stem cell fate: Wnt, notch, and hedgehog as the usual suspects. Curr Opin Hematol.

[49] Harasymiak-Krzyżanowska I, Niedojadło A, Karwat J, Kotuła L, Gil-Kulik P, Sawiuk M (2013). Adipose tissue-derived stem cells show considerable promise for regenerative medicine applications. Cell Mol Biol Lett..

[50] Houtgraaf J, den Dekker W, van Dalen B, Springeling T, de Jong R, van Geuns RJ (2012). First experience in humans using adipose tissue-derived regenerative cells in the treatment of patients with ST-segment elevation myocardial infarction. J Am Coll Cardiol.

[51] Garcia-Olmo D, Herreros D, Pascual I, Pascual JA, Del-Valle E, Zorrilla J (2009). Expanded adipose-derived stem cells for the treatment of complex perianal fistula: a phase II clinical trial. Dis Colon Rectum.

[52] Banyard DA, Salibian AA, Widgerow AD, Evans GR (2015). Implications for human adipose-derived stem cells in plastic surgery. J Cell Mol Med.

[53] Thesleff T, Lehtimäki K, Niskakangas T, Mannerström B, Miettinen S, Suuronen R (2011). Cranioplasty with adipose-derived stem cells and biomaterial: a novel method for cranial reconstruction. Neurosurgery..

[54] Sándor GK, Numminen J, Wolff J, Thesleff T, Miettinen A, Tuovinen VJ (2014). Adipose stem cells used to reconstruct 13 cases with cranio-maxillofacial hard-tissue defects. Stem Cell Transl Med.

[55] Galipeau J, Sensébé L (2018). Mesenchymal stromal cells: clinical challenges and therapeutic opportunities. Cell Stem Cell.

[56] Fang B, Song Y, Liao L, Zhang Y, Zhao RC (2007). Favorable response to human adipose tissue-derived mesenchymal stem cells in steroid-refractory acute graft-versus-host disease. Transplant Proc.

[57] Koh Y, Choi Y (2012). Infrapatellar fat pad-derived mesenchymal stem cell therapy for knee osteoarthritis. Knee..

[58] Mazini L, Rochette L, Amine M, Malka G (2019). Regenerative capacity of adipose derived stem cells (ADSCs), comparison with mesenchymal stem cells (MSCs). Int J Mol Sci.

[59] Mailey B, Hosseini A, Baker J, Young A, Alfonso Z, Hicok K (2014). Adipose-derived stem cells: methods for isolation and applications for clinical use. Methods Mol Biol.

[60] Jang YK, Kim M, Lee YH, Oh W, Yang YS, Choi SJ (2014). Optimization of the therapeutic efficacy of human umbilical cord blood-mesenchymal stromal cells in an NSG mouse xenograft model of graft-versus-host disease. Cytotherapy..

[61] Lee SH, Kim DS, Lee MW, Noh YH, Jang IK, Kim DH (2013). A strategy for enhancing the engraftment of human hematopoietic stem cells in NOD/SCID mice. Ann Hematol.

[62] Strioga M, Viswanathan S, Darinskas A, Slaby O, Michalek J (2012). Same or not the same? Comparison of adipose tissue-derived versus bone marrow-derived mesenchymal stem and stromal cells. Stem Cells Dev.

[63] Ivanova-Todorova E, Bochev I, Mourdjeva M, Dimitrov R, Bukarev D, Kyurkchiev S (2009). Adipose tissue-derived mesenchymal stem cells are more potent suppressors of dendritic cells differentiation compared to bone marrow-derived mesenchymal stem cells. Immunol Lett.

[64] Jin H, Bae Y, Kim M, Kwon SJ, Jeon HB, Choi SJ (2013). Comparative analysis of human mesenchymal stem cells from bone marrow, adipose tissue, and umbilical cord blood as sources of cell therapy. Int J Mol Sci.

[65] Li X, Bai J, Ji X, Li R, Xuan Y, Wang Y (2014). Comprehensive characterization of four different populations of human mesenchymal stem cells as regards their immune properties, proliferation and differentiation. Int J Mol Med.

[66] Bernardo ME, Fibbe WE (2015). Mesenchymal stromal cells and hematopoietic stem cell transplantation. Immunol Lett.

[67] Wen F, Zhang HJ, Chen Y, Yue Q, Liu Z, Zhang Q (2015). Sca1(+) mesenchymal stromal cells inhibit graft-versus-host disease in mice after bone marrow transplantation. Int Immunopharmacol.

[68] Le Blanc K, Frassoni F, Ball L, Locatelli F, Roelofs H, Lewis I (2008). Mesenchymal stem cells for treatment of steroid-resistant, severe, acute graft-versus-host disease: a phase II study. Lancet..

[69] Dander E, Lucchini G, Vinci P, Introna M, Masciocchi F, Perseghin P (2012). Mesenchymal stromal cells for the treatment of graft-versus-host disease: understanding the in vivo biological effect through patient immune monitoring. Leukemia..

[70] Jurado M, De La Mata C, Ruiz-García A, López-Fernández E, Espinosa O, Remigia MJ (2017). Adipose tissue-derived mesenchymal stromal cells as part of therapy for chronic graft-versus-host disease: a phase I/II study. Cytotherapy..

[71] Alluri R, Jakus A, Bougioukli S, Pannell W, Sugiyama O, Tang A (2018). 3D printed hyperelastic “bone” scaffolds and regional gene therapy: a novel approach to bone healing. J Biomed Mater Res A.

[72] Kim BS, Kwon YW, Kong JS, Park GT, Gao G, Han W (2018). 3D cell printing of in vitro stabilized skin model and in vivo pre-vascularized skin patch using tissue-specific extracellular matrix bioink: a step towards advanced skin tissue engineering. Biomaterials..

[73] Lee MK, DeConde AS, Lee M, Walthers CM, Sepahdari AR, Elashoff D (2015). Biomimetic scaffolds facilitate healing of critical-sized segmental mandibular defects. Am J Otolaryngol.

[74] Temple JP, Hutton DL, Hung BP, Huri PY, Cook CA, Kondragunta R (2014). Engineering anatomically shaped vascularized bone grafts with hASCs and 3D-printed PCL scaffolds. J Biomed Mater Res A.

[75] Van Harmelen V, Skurk T, Rohrig K, Lee YM, Halbleib M, Aprath-Husmann I (2003). Effect of BMI and age on adipose tissue cellularity and differentiation capacity in women. Int J Obes Relat Metab Disord.

[76] Di Taranto G, Cicione C, Visconti G, Isgrò MA, Barba M, Di Stasio E (2015). Qualitative and quantitative differences of adipose-derived stromal cells from superficial and deep subcutaneous lipoaspirates: a matter of fat. Cytotherapy..

[77] Dubey NK, Mishra VK, Dubey R, Deng YH, Tsai FC, Deng WP (2018). Revisiting the advances in isolation, characterization and secretome of adipose-derived stromal/stem cells. Int J Mol Sci.

[78] Jung S, Kleineidam B, Kleinheinz J (2015). Regenerative potential of human adipose-derived stromal cells of various origins. J Craniomaxillofac Surg.

[79] Baglioni S, Cantini G, Poli G, Francalanci M, Squecco R, Di Franco A (2012). Functional differences in visceral and subcutaneous fat pads originate from differences in the adipose stem cell. PLoS One.

[80] Van Harmelen V, Rohrig K, Hauner H (2004). Comparison of proliferation and differentiation capacity of human adipocyte precursor cells from the omental and subcutaneous adipose tissue depot of obese subjects. Metabolism..

[81] Bakker AH, Van Dielen FM, Greve JW, Adam JA, Buurman WA (2004). Preadipocyte number in omental and subcutaneous adipose tissue of obese individuals. Obes Res.

[82] Jurgens WJ, Oedayrajsingh-Varma MJ, Helder MN, Zandiehdoulabi B, Schouten TE, Kuik DJ (2008). Effect of tissue-harvesting site on yield of stem cells derived from adipose tissue: implications for cell-based therapies. Cell Tissue Res.

[83] Klar AS, Zimoch J, Biedermann T (2017). Skin tissue engineering: application of adipose-derived stem cells. Biomed Res Int.

[84] Santos Rizzo Zuttion MS, Dias Câmara DA, Dariolli R, Takimura C, Wenceslau C, Kerkis I (2019). In vitro heterogeneity of porcine adipose tissue-derived stem cells. Tissue Cell.

[85] Raposio E, Simonacci F, Perrotta RE (2017). Adipose-derived stem cells: Comparison between two methods of isolation for clinical applications. Ann Med Surg (Lond).

[86] Choi MR, Kim HY, Park JY, Lee TY, Baik CS, Chai YG (2010). Selection of optimal passage of bone marrow-derived mesenchymal stem cells for stem cell therapy in patients with amyotrophic lateral sclerosis. Neurosci Lett.

[87] Debnath T, Chelluri LK (2019). Standardization and quality assessment for clinical grade mesenchymal stem cells from human adipose tissue. Hematol Transfus Cell Ther.

[88] Wan Safwani WK, Makpol S, Sathapan S, Chua KH (2011). The changes of stemness biomarkers expression in human adipose-derived stem cells during long-term manipulation. Biotechnol Appl Biochem.

